# Long-term survival after targeted therapy plus immunotherapy without chemotherapy in advanced gallbladder carcinoma: a case report and literature review

**DOI:** 10.3389/fimmu.2025.1629985

**Published:** 2025-09-26

**Authors:** Zhitao Chen, Yuhao Wu, Chenchen Ding, Yangjun Gu, Weilin Wu, Qiyong Li

**Affiliations:** ^1^ Department of Hepatobiliary Surgery, Shulan (Hangzhou) Hospital Affiliated to Zhejiang Shuren University Shulan International Medical College, Hangzhou, China; ^2^ Women & Children Health Care Hospital of Quzhou, Quzhou, Zhejiang, China; ^3^ Affiliated Mental Health Centre & Hangzhou Seventh People’s Hospital, Zhejiang University School of Medicine, Hangzhou, Zhejiang, China

**Keywords:** gallbladder carcinoma, camrelizumab, apatinib, immunotherapy, case report

## Abstract

**Background:**

Gallbladder carcinoma (GBC) is a highly aggressive malignancy with limited treatment options and poor prognosis, particularly in advanced stages. Traditional chemotherapy often yields modest outcomes and can be poorly tolerated.

**Materials and methods:**

We report a case of a 59-year-old woman with stage IVA GBC who was unable to tolerate first-line gemcitabine and cisplatin due to severe adverse effects. Next-generation sequencing revealed a moderate tumor mutation burden and microsatellite-stable status. Based on a multidisciplinary team assessment, she received camrelizumab (PD-1 inhibitor) plus apatinib (VEGFR2 inhibitor).

**Results:**

After 11 cycles, imaging demonstrated significant tumor regression and reduced invasion, enabling successful radical resection. Postoperative pathology confirmed moderately differentiated adenocarcinoma with no lymph node involvement. The patient remained disease-free for over two years. Following isolated cervical lymph node metastasis, combination therapy with camrelizumab and surufatinib resulted in complete remission. She continues on maintenance immunotherapy without recurrence five years post-diagnosis.

**Conclusion:**

This case highlights the promising potential of targeted therapy and immunotherapy in converting unresectable GBC to operable disease and achieving durable remission, even in patients lacking classic immunotherapy biomarkers. Personalized, non-chemotherapy-based strategies may offer viable alternatives for selected patients.

## Introduction

Gallbladder carcinoma (GBC), accounting for approximately 80–90% of biliary tract cancer (BTC) cases, ranks sixth among malignancies of the digestive system (1, 2). In 2020, global estimates from the International Agency for Research on Cancer (IARC) reported approximately 115,900 new cases of GBC and 85,000 related deaths (2). GBC is frequently asymptomatic in its early stages, leading to delayed diagnosis and a high likelihood of presentation at advanced, less treatable stages (3). Advanced GBC is characterized by aggressive behavior and an unfavorable prognosis, with a median overall survival (mOS) of approximately 6 months and a 5-year survival rate below 10% (4). For patients with resectable GBC, surgical resection remains the only potentially curative treatment (5, 6). However, in cases of unresectable or metastatic GBC, the prognosis is poor, as curative surgery is not feasible and current management primarily consists of palliative care due to the limited effectiveness of conventional chemotherapy (7, 8). Historically, first-line therapies for unresectable GBC have included combinations such as gemcitabine plus cisplatin (GC), gemcitabine with S-1 (GS), and the triplet regimen of gemcitabine, cisplatin, and S-1 (GCS) (7–9). Despite the expansion of systemic treatment options, achieving conversion to resectable disease remains a significant challenge.

In recent years, the combination of targeted therapy and immunotherapy has transformed the management of solid tumors, resulting in significant gains in patient survival outcomes. In BTC, recent progress in chemo-immunotherapy—combining chemotherapy with immunotherapy—has demonstrated superior efficacy over traditional regimens, leading to improved survival and response rates in patients with metastatic cholangiocarcinoma. Clinical trials such as TOPAZ-1 and KEYNOTE-966 have investigated the therapeutic impact of combining chemotherapy with immune checkpoint inhibitors—durvalumab and pembrolizumab, respectively—in patients with intrahepatic and extrahepatic cholangiocarcinoma, as well as GBC (10, 11). Findings from the TOPAZ-1 trial show that treatment with gemcitabine, cisplatin, and durvalumab significantly improved mOS (12.8 vs. 11.5 months), 24-month OS rate (24.9% vs. 10.4%), and progression-free survival (7.2 vs. 5.7 months) compared to chemotherapy alone, without an increase in adverse events (10). In December 2022, this combination regimen received approval in Japan for the treatment of unresectable biliary tract cancer. However, no established regimen currently exists for the use of immunotherapy or targeted therapy—either alone or in combination—specifically for GBC.

Here, we present a case of locally advanced GBC that responded remarkably well to combined targeted therapy and immunotherapy, enabling successful conversion surgery and resulting in long-term favorable outcomes. This case highlights the potential of emerging therapeutic strategies to improve outcomes in GBC, a malignancy traditionally associated with poor prognosis. In parallel, comprehensive analysis integrating targeted gene sequencing with conventional histopathology was conducted to explore the patient’s mutational landscape, regulatory mechanisms, functional interactions, and enriched signaling pathways. Given the distinct biological and clinical features of GBC, evaluating treatment efficacy in this specific context is essential. A deeper understanding of these complexities may help refine therapeutic approaches and enhance clinical outcomes.

## Ethical approval and patient consent

This case report was prepared in accordance with the CARE (CAse REport) guidelines. Written informed consent was obtained from the patient for the publication of this report and any accompanying images. The study protocol was reviewed and approved by the Ethics Committee of Shulan (Hangzhou) Hospital.

## Case report

**Figure 1:**
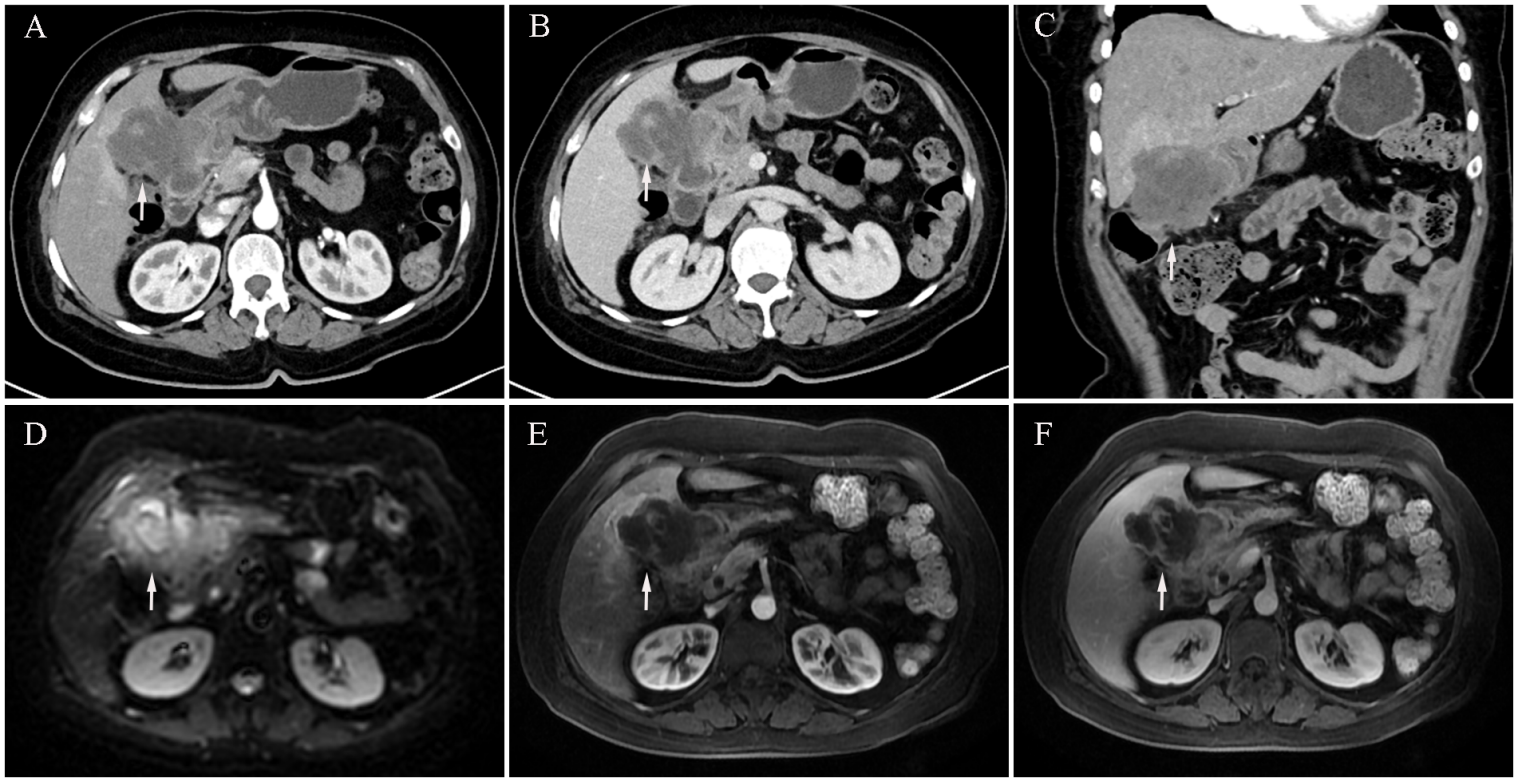
Initial contrast-enhanced abdominal computed tomography (CT) and Upper abdominal dynamic contrast enhanced magnetic resonance imaging (MRI) at diagnosis (March 25, 2020). **(A)** CT arterial phase showing marked heterogeneous enhancement of the gallbladder lesion (white arrow, longest diameter was 73 mm). **(B)** CT venous phase showing marked heterogeneous enhancement of the gallbladder lesion (white arrow). **(C)** Coronal CT image showing gallbladder lesion invading adjacent liver tissue, gastric antrum, and hepatic flexure of the colon (white arrow). **(D)** MRI diffusion weighted imaging (DWI) sequence shows mixed high signal intensity with poorly defined tumor margins (white arrow). **(E)** MRI arterial sequence shows marked heterogeneous enhancement of the gallbladder lesion (white arrow). **(F)** MRI venous sequence showing marked heterogeneous enhancement of the gallbladder lesion (white arrow).

We report the case of a 59-year-old woman who presented with a two-month history of intermittent right upper quadrant pain that was mild, self-limiting, and typically resolved within a few hours. She was admitted to Shulan (Hangzhou) Hospital on March 25, 2020. She denied associated symptoms such as fever, nausea, vomiting, or diarrhea, and had no notable past medical history. On admission, physical examination revealed a firm mass approximately 3 cm in size palpable beneath the right costal margin, corresponding to the liver edge, with mild tenderness elicited on palpation. Laboratory evaluation revealed a mildly elevated white blood cell count of 11.1×10^9^/L (reference range: 3.5–9.5×10^9^/L), a C-reactive protein level of 44.5 mg/L (normal ≤10 mg/L), and a platelet count of 372×10^9^/L (reference range: 125–350×10^9^/L). Fecal occult blood testing returned a positive result. Serum tumor markers were markedly elevated, including carcinoembryonic antigen (CEA) at 24.5 ng/mL, carbohydrate antigen (CA)19–9 at 48.7 U/mL, cytokeratin 19 fragment (CYFRA 21-1) at 9.2 ng/mL, CA72–4 at 8.4 IU/mL, and CA125 at 113.5 U/mL. Contrast-enhanced abdominal computed tomography (CT) revealed an ill-defined, irregular hypodense mass in the gallbladder fossa measuring approximately 7.3 × 6.4 cm, with heterogeneous gallbladder wall thickening and marked, uneven enhancement (**Figures 1A–C**). The lesion involved adjacent hepatic tissue, the gastric antrum, and the hepatic flexure of the colon, with indistinct margins near the greater omentum (**Figures 1A–C**). Upper abdominal dynamic contrast enhanced magnetic resonance imaging (MRI) showed the mass as slightly hyperintense on T1-weighted images, heterogeneously hyperintense on T2, and exhibited mixed high signal intensity on diffusion weighted imaging (DWI) (**Figures 1D–F**). Gastrointestinal endoscopy further confirmed malignant invasion of the descending duodenum and transverse colon (**Supplementary Figures S1A, B**). Biopsy specimens were obtained, and histopathological analysis confirmed adenosquamous carcinoma (**Supplementary Figure S1C**). According to the TNM classification system of the American Joint Committee on Cancer (AJCC), a definitive diagnosis of primary gallbladder carcinoma (T4N1M0, Stage IVA) was established based on the clinical and pathological findings.

Given that the gemcitabine and cisplatin combination is the established first-line regimen, it has been widely adopted as the standard adjuvant therapy for advanced GBC. On April 2, 2020, the patient initiated treatment with a gemcitabine and cisplatin regimen, consisting of gemcitabine at 1000 mg/m² and cisplatin at 25 mg/m² administered on days 1 and 8 of a three-week cycle. Unfortunately, on the third day following the first chemotherapy cycle, the patient developed fever, oral mucosal ulcerations with purulent discharge, visual impairment in both eyes, and erythematous rashes on the hands and feet. A multidisciplinary team (MDT) consultation resulted in a clinical diagnosis of Stevens-Johnson syndrome. Unfortunately, the patient developed gastrointestinal obstruction, which was managed by endoscopic placement of a jejunal feeding tube to initiate enteral nutrition (**Supplementary Figure S1D**). Concurrently, symptomatic and supportive therapy with methylprednisolone and intravenous immunoglobulin was administered, leading to gradual clinical improvement. These adverse events not only diminished the patient’s quality of life but also led to refusal of further chemotherapy. To explore additional therapeutic options, the patient underwent next-generation sequencing (NGS) using a panel covering 688 genes and 15 microsatellite loci (performed on the MGISEQ-2000 platform). The analysis identified a tumor mutation burden (TMB) of 7.89 mutations/Mb and a microsatellite-stable (MSS) status. We summarized the somatic mutation patterns and mutation frequencies identified in the patient’s genetic testing results (**Supplementary Table S1**). A MDT discussion was reconvened, considering the growing evidence supporting the efficacy of targeted therapy and immunotherapy—including in GBC—with relatively manageable toxicity profiles (12–17). Moreover, the elevated TMB may suggest a potential benefit from immunotherapy. Considering the patient’s financial situation and the previously demonstrated efficacy of camrelizumab combined with apatinib in biliary tract malignancies, a treatment regimen was initiated after obtaining informed consent. The patient received camrelizumab (Jiangsu Hengrui Pharmaceuticals Co., Ltd.) intravenously at a dose of 200 mg every three weeks, along with oral apatinib (Jiangsu Hengrui Pharmaceuticals Co., Ltd.) at 250 mg once daily in 4-week cycles.

**Figure 2:**
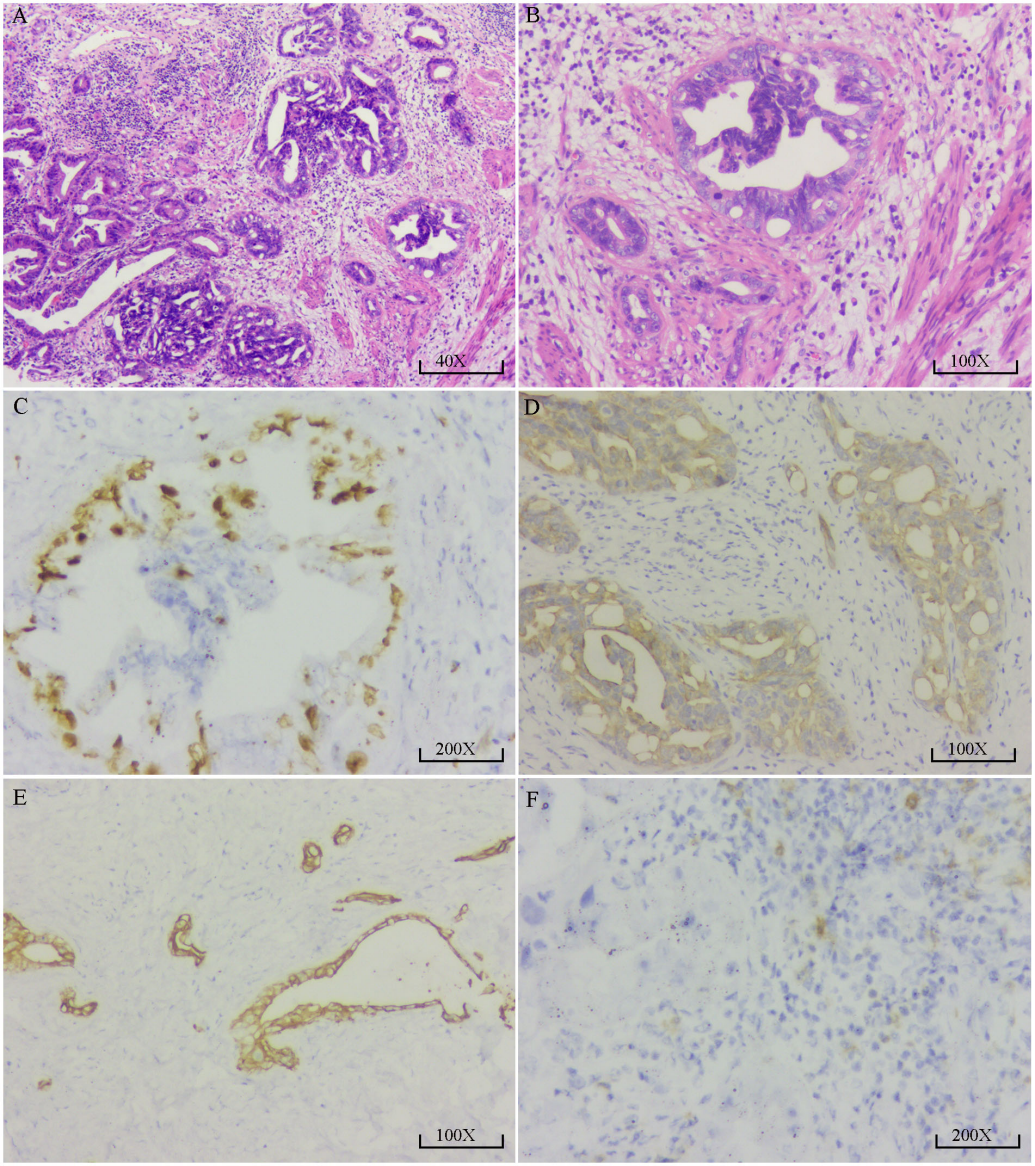
Pathological and immunohistochemical findings of the resected gallbladder carcinoma. **(A)** High-power H&E staining (40×) shows irregular proliferation of glandular structures with disorganized arrangement. Some glandular lumens appear cribriform. The tumor cells exhibit moderate to high-grade atypia, enlarged hyperchromatic nuclei with visible nucleoli, and stromal infiltration by lymphocytes, consistent with moderately to poorly differentiated adenocarcinoma. **(B)** H&E staining at higher magnification (100×) reveals irregular glandular architecture with dilated and abnormally shaped lumens. The glandular epithelial cells are arranged in a pseudostratified pattern with deeply stained, pleomorphic nuclei and occasional nucleoli. Stromal fibrosis with lymphocytic infiltration is also noted. **(C)** Immunohistochemical staining for Ki-67 (200×) shows brown nuclear positivity in tumor cells, indicating high proliferative activity. Positive staining is mainly observed in the epithelial layer of the glands, with stronger intensity in some areas; approximately 30–40% of tumor cells are positive. **(D)** CK19 immunostaining (100×) reveals strong brown cytoplasmic and membranous positivity in tumor cells, predominantly localized to the epithelial cells of glandular structures. The stromal background shows no staining, indicating high specificity. **(E)** CK7 immunostaining (100×) demonstrates diffuse brown cytoplasmic positivity in tumor cells forming gland-like structures. The staining is distinct and widespread, with a clear negative background. **(F)** PD-1 immunostaining (200×) shows brown granular staining in a subset of stromal lymphocytes, suggesting low to moderate PD-1 expression in tumor-infiltrating T cells. Tumor cells themselves are negative for PD-1 expression.

After four cycles of combined targeted and immunotherapy, contrast-enhanced abdominal CT imaging revealed a marked reduction in the size and enhancement of the GBC, as well as decreased invasion into adjacent liver tissue, the gastric antrum, and the hepatic flexure of the colon compared to previous scans (**Supplementary Figure S2A–C**). Given that R0 resection was not feasible at the time, the patient continued on combined targeted and immunotherapy until December 2020 (a total of 11 cycles). No significant adverse reactions were observed during the patient’s targeted and immunotherapy treatment. Follow-up contrast-enhanced CT demonstrated ongoing hepatic involvement; however, the primary tumor had significantly decreased in size, and no evidence of invasion into surrounding structures, including the gastric antrum and hepatic flexure of the colon, was observed (**Supplementary Figures S2D–F**). At the same time, the tumor marker CA19–9 level returned to the normal range (**Supplementary Figure S2G**). On December 3, 2020, the patient underwent exploratory laparotomy. A fibrotic mass approximately 1.5 cm in size was identified at the gallbladder fundus, with no apparent tumor invasion or metastasis observed in the right colonic flexure or the second portion of the duodenum. Lymph nodes #7, #8, #9, #12, and #13 were dissected, and intraoperative frozen section analysis confirmed a negative biliary margin. Comprehensive postoperative histopathological analysis confirmed moderately differentiated adenocarcinoma of the gallbladder, with no lymph node metastasis detected (0/9) (**Figures 2A, B**). Immunohistochemistry showed positive staining for CA19-9, CEA, CK19, CK20, CK7, MLH1, MSH2, MSH6, PD-1 (2%), and Ki-67 (30%) (**Figures 2C–F**). The postoperative recovery was uneventful, and the patient was discharged on day 16. She continued adjuvant therapy with camrelizumab every three weeks in combination with apatinib and remained disease-free for two years following surgery.

**Figure 3:**
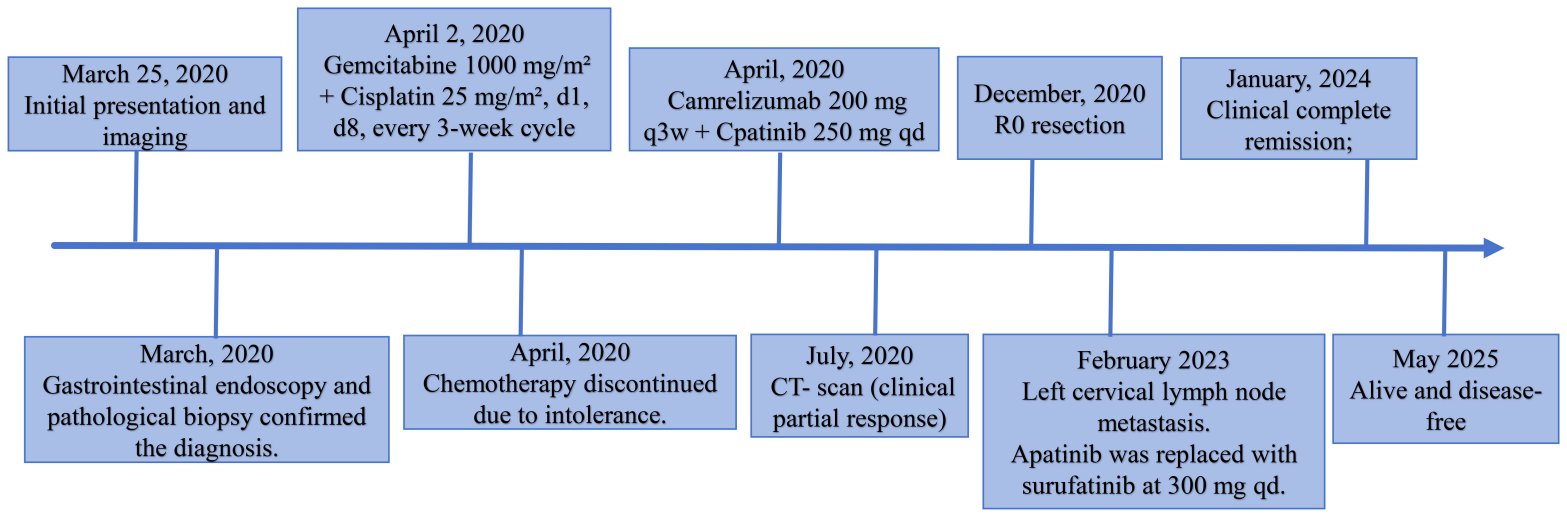
The timeline of the entire clinical course.

In February 2023, more than two years after surgery, whole-body ^18^F-fluorodeoxyglucose (FDG) PET/CT was performed to assess the necessity of continuing adjuvant therapy with camrelizumab and apatinib. Unexpectedly, an enlarged lymph node measuring approximately 0.5 cm was detected in the left cervical region, with a maximum standardized uptake value (SUVmax) of 3.37, raising suspicion for metastatic disease. To confirm the suspected diagnosis, ultrasound-guided fine-needle aspiration biopsy of the cervical lymph node was performed. Histopathological analysis confirmed the presence of metastatic carcinoma. Following a comprehensive review of the patient’s clinical history and a renewed MDT discussion, the treatment plan was adjusted by replacing apatinib with surufatinib (Hutchmed, Shanghai) at 300 mg once daily in 4-week cycles. Interestingly, after 15 cycles of combination therapy, the cervical metastatic lesion gradually regressed and eventually resolved completely. The patient is currently receiving maintenance therapy with camrelizumab alone, with no evidence of recurrence or metastasis. Remarkably, five years have passed since the diagnosis of locally advanced GBC, and the patient remains alive and disease-free. A summary of the clinical course and treatments is presented in the timeline depicted in **Figure 3**.

## Discussion

GBC is a highly aggressive malignancy with limited treatment efficacy, largely due to its poor responsiveness to chemotherapy (18, 19).Despite continuous research and clinical efforts, therapeutic advancements for GBC have remained limited over the years (20, 21). The TOPAZ-1 trial offers a glimmer of hope in the treatment of BTC; however, the objective response rate (ORR) for the GC regimen was 26.7%, which was notably lower than the 41% reported in the MITSUBA trial with the GCS regimen, suggesting limited efficacy in achieving significant local tumor regression (9, 10). At present, the survival benefit of targeted therapy combined with immunotherapy, when used without chemotherapy, remains unclear in patients with GBC, as this approach has been rarely explored in prior studies. In the present case, treatment with camrelizumab and apatinib led to a clinical partial response (PR) in a patient with locally advanced GBC, resulting in significant tumor reduction and successful conversion to radical resection. This case highlights the potential clinical value of this therapeutic strategy.

The advent of checkpoint inhibitors and targeted therapies has sparked increasing interest in combination immunotherapy strategies for cancer treatment, an approach that has also gained traction in the management of GBC (22, 23). A growing body of evidence indicates that immune checkpoint inhibitors (ICIs) play a significant role in advancing the treatment of BTC. In a phase II trial, Feng et al (24). reported that the combination of nivolumab with GC demonstrated notable antitumor efficacy and acceptable safety in patients with unresectable or metastatic BTC, supporting further investigation of this chemo-immunotherapy approach. In their study, 15 patients (55.6%) achieved an objective response, including 5 (18.6%) with a complete response (CR), and the disease control rate (DCR) reached 92.6%. Rimini et al (25). conducted the first real-world multicenter study showing that the combination of durvalumab with GC as first-line treatment for advanced BTC yielded survival outcomes and safety comparable to those reported in the phase III TOPAZ-1 trial, reinforcing its applicability in routine clinical settings. The confirmed ORR was 34.5%, and the DCR reached 87.6%. Regrettably, none of the aforementioned studies included subgroup analyses focused specifically on GBC. Therefore, further investigation is required to assess the therapeutic efficacy of combining immunotherapy or targeted therapy with the GC regimen specifically in patients with GBC. In the field of targeted and immunotherapy for GBC, Tan et al (26). reported that a combination of anti-PD-1 antibodies, lenvatinib, and GEMOX chemotherapy as a non-first-line treatment demonstrated promising clinical efficacy and acceptable safety in patients with advanced GBC. The regimen achieved a mOS of 15.1 months and a DCR of 75%, supporting its potential as a viable option for subsequent-line therapy.

Although targeted and immunotherapy have been applied sporadically in the clinical management of GBC, their broader use remains limited. This is largely due to the high pathological heterogeneity of GBC, its complex molecular landscape lacking well-defined driver mutations, and characteristics of the tumor microenvironment—such as poor T-cell infiltration and limited immune activation—classifying it as a “cold tumor.” These factors collectively hinder the effectiveness and widespread implementation of targeted and immune-based therapies in GBC. To gain a clearer understanding of the clinical application of targeted and immunotherapy in gallbladder carcinoma, we conducted a comprehensive review and analysis of case reports indexed in PubMed. Our literature search identified 20 publications between 2019 and 2025 reporting on 27 patients with advanced gallbladder carcinoma who received immunotherapy or combined targeted therapy alongside conventional chemotherapy (27–46) (**Supplementary Table S2**). These reports collectively suggest that, despite the historically poor prognosis of advanced GBC, meaningful treatment responses can still be achieved. Personalized treatment approaches—especially those integrating targeted and immunotherapy—have shown encouraging potential in enhancing clinical outcomes, even in advanced or metastatic settings. Both the case reported by Prieto et al (28). and our current case describe patients with advanced GBC who, following successful conversion therapy, underwent curative resection and achieved survival beyond five years. Notably, in our case, the patient was unable to tolerate chemotherapy and was treated exclusively with a combination of targeted therapy and immunotherapy. To advance personalized treatment and improve long-term outcomes in GBC, this study proposes several key recommendations.

Considering the molecular heterogeneity of GBC, comprehensive genomic profiling should be strongly recommended in all cases of advanced disease. Detecting actionable genetic alterations expands potential treatment avenues and supports a more tailored, precision-based therapeutic strategy. HER2-positive gallbladder cancer has demonstrated notable responses to trastuzumab-based therapies, with enhanced efficacy observed when combined with PD-1 blockade such as camrelizumab (28, 36). Additionally, MET amplification, though less common, represents a potentially actionable alteration in gallbladder cancer, with cases showing clinical response to MET inhibitors like crizotinib following chemotherapy failure (31). To better inform clinical decision-making, we summarized the current molecular targets relevant to GBC and their associated mutation frequencies, along with corresponding therapeutic recommendations (**Table 1**).

**Table 1:** Common molecular alterations in gallbladder carcinoma and corresponding targeted therapy recommendations.

Druggable molecular alterations	Variation frequency	Recommended drugs
FGFR2 fusion/ rearrangement	0-3.0%	Pemigatinib
*IDH1*-mutations	0-1.7%	Ivosidenib
BRAF mutations	1%-5.9%	DabTram (Dabrafenib and Trametinib)
ERBB2 mutations/overexpression	8.3%-16.0%	Trastuzumab Deruxtecan
NTRK1-3 fusion/ rearrangement	1.7%	Larotrectinib/ entrectinib
RET fusion/ rearrangement	–	Pralsetinib/ Selpercatinib
KRAS-G12C mutations	0-2.3%	Adagrasib
PTEN mutations	0-57%	Bortezomib
MET overexpression/ amplification	39.8%/18.3%	Crizotinib

FGFR2: Fibroblast Growth Factor Receptor 2, IDH1: Isocitrate Dehydrogenase 1, BRAF: B-Raf Proto-Oncogene, Serine/Threonine Kinase, ERBB2: Erb-B2 Receptor Tyrosine Kinase 2 , NTRK1-3: Neurotrophic Receptor Tyrosine Kinase 1 / 2 / 3, RET: RET Proto-Oncogene, Receptor Tyrosine Kinase, KRAS-G12C: KRAS Proto-Oncogene, GTPase (specific mutation at codon 12: Glycine to Cysteine), PTEN: Phosphatase and Tensin Homolog, MET: MET Proto-Oncogene, Receptor Tyrosine Kinase.

Although PD-L1 expression, microsatellite instability (MSI), and high tumor mutational burden (TMB-H) are recognized biomarkers predictive of response to immunotherapy, their absence should not preclude the use of immunotherapy in gallbladder carcinoma. Our findings suggest that patients may still derive clinical benefit from immunotherapy even when these biomarkers are negative. Li et al (29). reported a case of advanced GBC with low PD-L1 expression (5%) and MSI, in which treatment with pembrolizumab combined with GEMOX chemotherapy resulted in sustained clinical benefit. The patient achieved a partial response that progressed to a CR, followed by stable disease maintained for over 14 months. Rao et al. also reported a case of advanced GBC with weak PD-L1 expression (10%), MSS, and low tumor mutational burden (TMB-L), in which combination therapy with camrelizumab and apatinib led to a CR after five cycles, highlighting the potential benefit of immunotherapy even in biomarker-low patients.

Our review highlights the potential application of combination strategies integrating chemotherapy with targeted agents and ICIs across multiple clinical contexts. These include neoadjuvant settings to enable surgical resection, adjuvant therapy after palliative procedures, and treatment of postoperative recurrence. Prieto et al (28). described a case of stage IV GBC with liver and distant lymph node metastases that was successfully downstaged using trastuzumab combined with chemotherapy, enabling curative-intent resection and resulting in over five years of recurrence-free survival. Yi et al. reported a stage IVB GBC patient who underwent resection of the primary tumor followed by first-line chemo-immunotherapy (S1 plus pembrolizumab) for unresectable lymph node metastases, achieving a CR that has been maintained for over 32 months.

Elderly patients with GBC require special clinical consideration due to reduced physiological reserve and limited tolerance to intensive chemotherapy. For this population, lower-toxicity regimens—such as S-1 in combination with targeted therapy and immunotherapy—may offer a more suitable alternative, maintaining therapeutic efficacy while minimizing adverse effects and enhancing treatment adherence and quality of life. Zhang et al (39). reported that in five cases of advanced GBC, the combination of tislelizumab and the low-toxicity oral fluoropyrimidine S-1 achieved sustained clinical responses, including long-term disease control and complete remission in selected patients. In cases where chemotherapy is not feasible, dual targeted therapy and immunotherapy may serve as an effective treatment strategy. Although the use of targeted therapy in combination with immunotherapy alone has shown limited efficacy in GBC, its potential role warrants further investigation within carefully selected patient populations (23). However, the present case illustrates this approach. An advanced GBC patient, ineligible for cytotoxic chemotherapy, achieved a notable clinical response with a combination of a tyrosine kinase inhibitor and an immune checkpoint inhibitor. Our findings are consistent with those of Zhong et al., who reported a recurrent pMMR/MSS GBC case achieving complete response to camrelizumab plus apatinib (TMB 7.26 mut/Mb; PD-L1 TPS 10%, IPS 20%). Together, these observations highlight the importance of adaptable, patient-centered treatment strategies and reflect the ongoing evolution of the therapeutic landscape for GBC.

This report is limited by its single-case design, which inherently restricts the generalizability of the findings. The favorable response to camrelizumab and apatinib may have been influenced by patient-specific factors, including genetic background and tumor microenvironment. Moreover, although the patient derived clinical benefit despite lacking classical immunotherapy biomarkers, the absence of detailed immune profiling and mechanistic investigations prevents the identification of potential predictive markers. Future large-scale studies are warranted to validate this chemotherapy-free combination strategy and to better define patient subgroups who may benefit from such individualized treatment approaches in advanced GBC.

## Conclusion

This case report demonstrates the potential efficacy of combined targeted therapy and immunotherapy in the management of locally advanced GBC, particularly in patients who are unable to tolerate conventional chemotherapy. Despite the historically poor prognosis associated with advanced GBC, our patient achieved significant tumor regression, successful conversion surgery, and long-term disease-free survival following a regimen of camrelizumab and apatinib. The clinical response observed, even in the absence of classical predictive biomarkers such as high PD-L1 expression or MSI, highlights the promise of individualized treatment strategies beyond standard chemotherapeutic approaches.

## Data Availability

The original contributions presented in the study are included in the article/**Supplementary Material**. Further inquiries can be directed to the corresponding authors.

## Supplementary material

Supplementary Figure 1 Endoscopic examination and biopsy findings. **(A)** Gastroscopy revealed tumor invasion of the gastric antrum, resulting in partial gastrointestinal obstruction. **(B)** Colonoscopy showed tumor infiltration at the hepatic flexure of the colon. **(C)** Biopsy of the gastric antrum lesion via gastroscopy confirmed a pathological diagnosis of adenocarcinoma. **(D)** Gastrointestinal contrast study demonstrated obstruction, with impaired passage of contrast agent.

Supplementary Figure 2 Contrast-enhanced abdominal computed tomography (CT) after 4 treatment cycles and after 11 cycles (Dec,2020, at the time of surgery). **(A)** After 4 treatment cycles, arterial phase CT shows significant reduction in gallbladder tumor size compared to baseline, with heterogeneous enhancement. **(B)** After 4 treatment cycles, venous phase CT demonstrates marked tumor shrinkage with persistent heterogeneous enhancement (longest diameter was 35 mm). **(C)** Coronal CT after 4 cycles shows a noticeable decrease in tumor size; however, invasion of the hepatic flexure and gastric antrum persists. **(D)** After 11 treatment cycles, arterial phase CT reveals no obvious gallbladder mass, and no heterogeneous enhancement is observed in the surrounding liver tissue. **(E)** After 11 treatment cycles, venous phase CT shows the tumor is no longer clearly visible, with no abnormal enhancement in adjacent liver tissue. **(F)** Coronal CT after 11 cycles indicates substantial tumor shrinkage compared to the 4-cycle scan, with minimal adhesion to the hepatic flexure and gastric antrum, but no clear evidence of invasion. **(G)** Changes in CA19–9 levels during treatment and over time.

Supplementary Figure 3 Cervical lymph node metastasis: PET-CT localization and pathological diagnosis. **(A)** The metastatic cervical lymph node shows a maximum SUV value of 3.37. **(B)** Axial view showing the anatomical location of the metastatic lesion. **(C)** Coronal view illustrating the position of the metastatic lymph node. **(D)** Pathological findings of the metastatic lymph node from fine-needle aspiration, magnification 200×. **(E)** Pathological findings of the metastatic lymph node from fine-needle aspiration, magnification 400×.
