# Supplementary figures and images for: Long-term survival after targeted therapy plus immunotherapy without chemotherapy in advanced gallbladder carcinoma: a case report and literature review
Source: Front Immunol. 2025 Sep 26;16:1629985. doi: 10.3389/fimmu.2025.1629985 (PMC12510835; doi:10.3389/fimmu.2025.1629985)

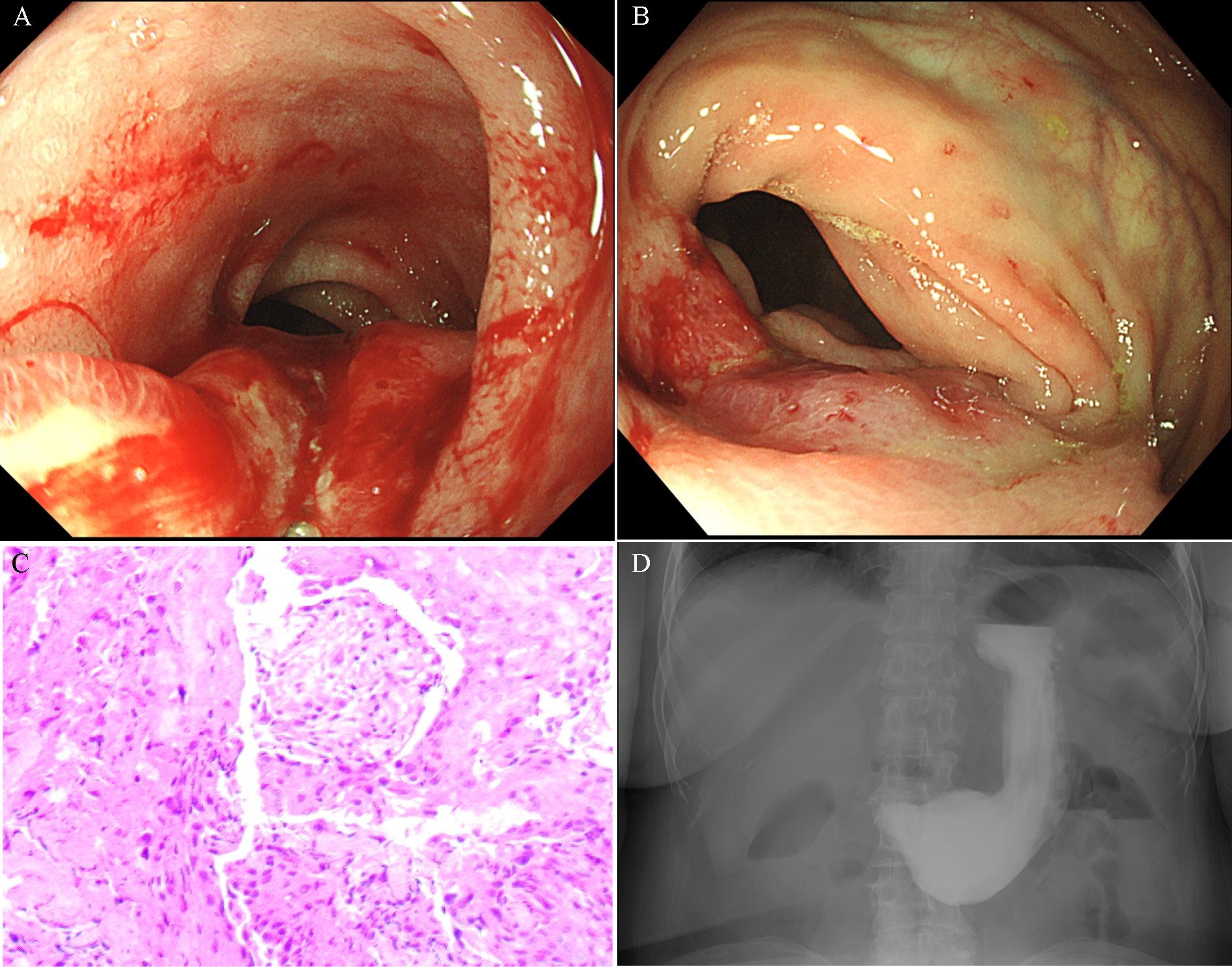

Supplement: Supplementary Figure 1 — Endoscopic examination and biopsy findings. (A) Gastroscopy revealed tumor invasion of the gastric antrum, resulting in partial gastrointestinal obstruction. (B) Colonoscopy showed tumor infiltration at the hepatic flexure of the colon. (C) Biopsy of the gastric antrum lesion via gastroscopy confirmed a pathological diagnosis of adenocarcinoma. (D) Gastrointestinal contrast study demonstrated obstruction, with impaired passage of contrast agent. [file Image1.tif]

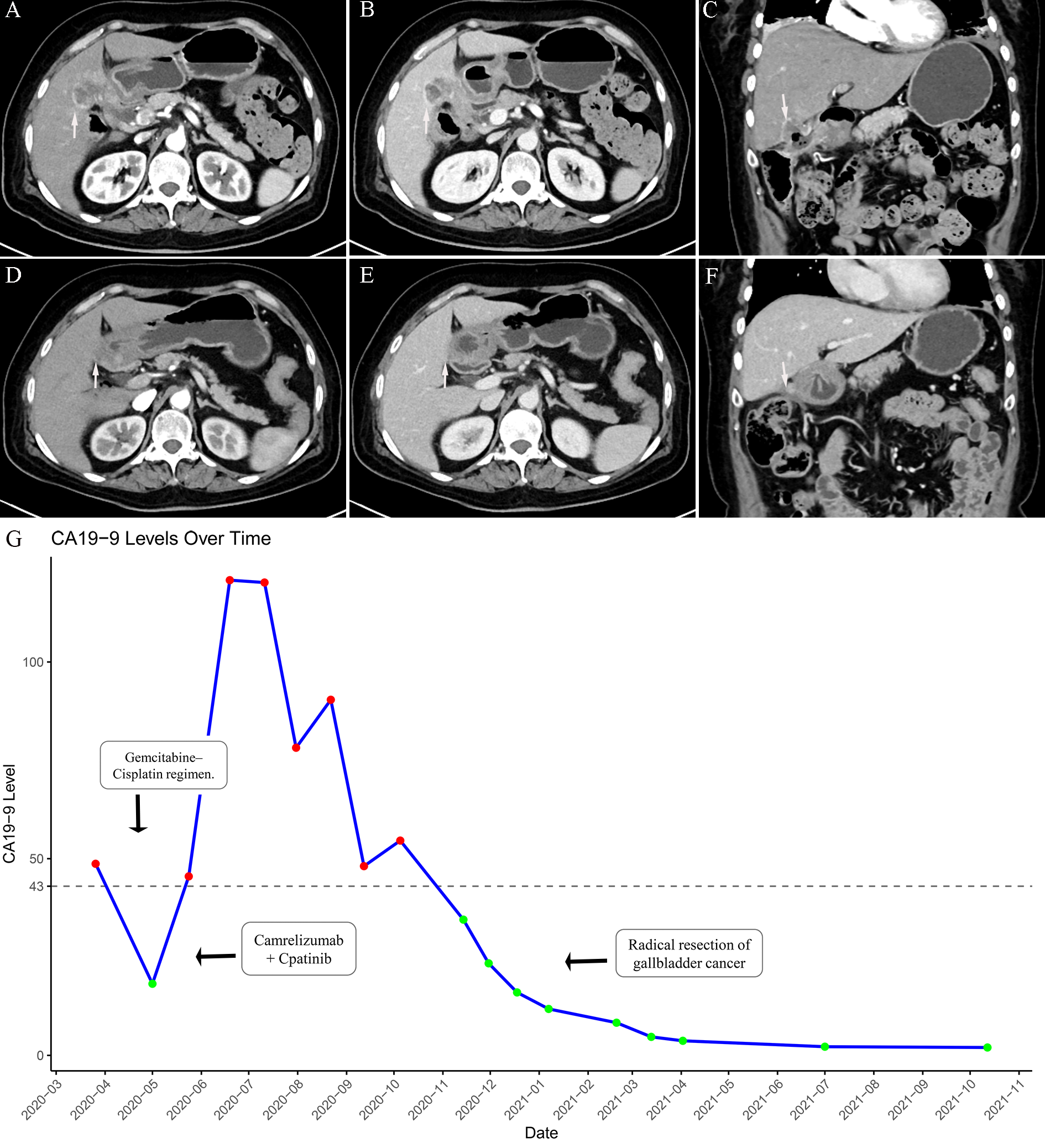

Supplement: Supplementary Figure 2 — Contrast-enhanced abdominal computed tomography (CT) after 4 treatment cycles and after 11 cycles (Dec,2020, at the time of surgery). (A) After 4 treatment cycles, arterial phase CT shows significant reduction in gallbladder tumor size compared to baseline, with heterogeneous enhancement. (B) After 4 treatment cycles, venous phase CT demonstrates marked tumor shrinkage with persistent heterogeneous enhancement (longest diameter was 35 mm). (C) Coronal CT after 4 cycles shows a noticeable decrease in tumor size; however, invasion of the hepatic flexure and gastric antrum persists. (D) After 11 treatment cycles, arterial phase CT reveals no obvious gallbladder mass, and no heterogeneous enhancement is observed in the surrounding liver tissue. (E) After 11 treatment cycles, venous phase CT shows the tumor is no longer clearly visible, with no abnormal enhancement in adjacent liver tissue. (F) Coronal CT after 11 cycles indicates substantial tumor shrinkage compared to the 4-cycle scan, with minimal adhesion to the hepatic flexure and gastric antrum, but no clear evidence of invasion. (G) Changes in CA19–9 levels during treatment and over time. [file Image2.tif]

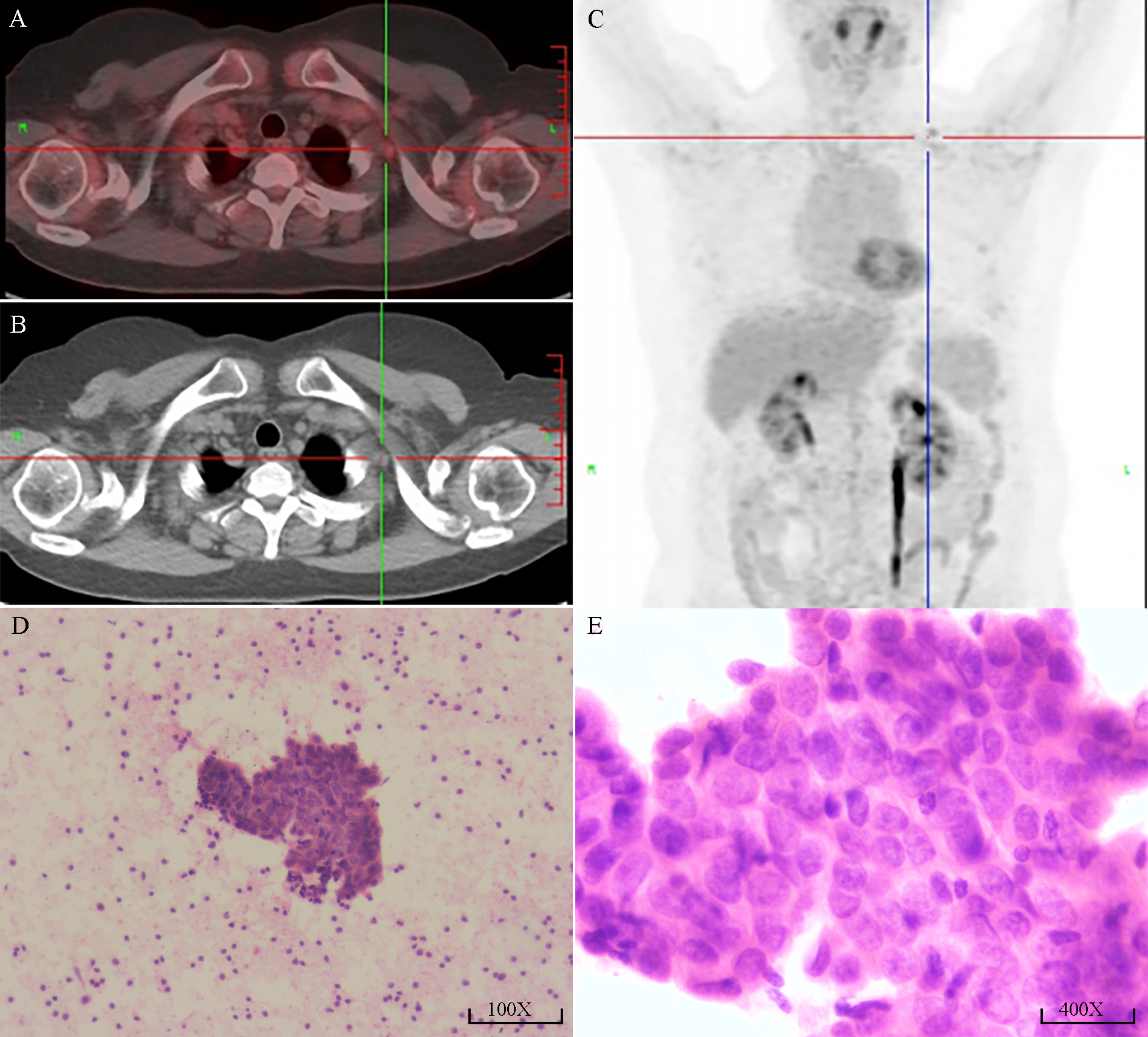

Supplement: Supplementary Figure 3 — Cervical lymph node metastasis: PET-CT localization and pathological diagnosis. (A) The metastatic cervical lymph node shows a maximum SUV value of 3.37. (B) Axial view showing the anatomical location of the metastatic lesion. (C) Coronal view illustrating the position of the metastatic lymph node. (D) Pathological findings of the metastatic lymph node from fine-needle aspiration, magnification 200×. (E) Pathological findings of the metastatic lymph node from fine-needle aspiration, magnification 400×. [file Image3.tif]
