# Supplementary material for: Long-term survival after targeted therapy plus immunotherapy without chemotherapy in advanced gallbladder carcinoma: a case report and literature review
Source: Front Immunol. 2025 Sep 26;16:1629985. doi: 10.3389/fimmu.2025.1629985 (PMC12510835; doi:10.3389/fimmu.2025.1629985)
Supplement: Supplementary file 4 [file Table1.docx]

**Table S1. Summary of somatic mutation patterns and mutation frequencies identified in the patient’s genetic testing results.**

| Gene | Test Result | Mutation Type | Transcript | Mutation Abundance (%) |
| --- | --- | --- | --- | --- |
| NF1 | p.E291* (c.871G>T) | Nonsense Mutation | NM_000267.3 | 56.59% |
| TP53 | p.Q38* (c.112C>T) | Nonsense Mutation | NM_000546.5 | 43.19% |
| ERBB2 | p.S310Y (c.929C>A) | Missense Mutation | NM_004448.2 | 12.2% |
| EGFR | Amplification | Amplification | NM_005228.3 | 18.99% |
| FBXW7 | Amplification | Amplification | NM_033632.3 | 1.18% |
| CDKN2A | Amplification | Amplification | NM_000077.4 | 3.11% |
| ELF3 | p.P294Hfs*62 (c.273_276delCTTC) | Frameshift Mutation | NM_001114309.1 | 48.18% |
| LATS1 | p.E753* (c.2259T>G) | Nonsense Mutation | NM_004690.3 | 37.11% |
| MYD88 | p.S279Afs*3 (c.834delC) | Frameshift Mutation | NM_002468.4 | 24.16% |
| SETD2 | p.Q2207* (c.6619C>T) | Nonsense Mutation | NM_014159.6 | 14.75% |
| SYK | p.E523K (c.1567G>A) | Missense Mutation | NM_003177.5 | 59.64% |
| JAK1 | p.N438S (c.1313A>G) | Missense Mutation | NM_002227.2 | 44.03% |
| SDHAF2 | p.R67T (c.200G>C) | Missense Mutation | NM_017841.2 | 37.83% |
| MUC16 | p.E1840Q (c.5410G>C) | Missense Mutation | NM_024690.2 | 33.01% |
| SHQ1 | p.E235K (c.703G>A) | Missense Mutation | NM_018130.2 | 30.71% |
| APC | p.R1523T (c.4568G>C) | Missense Mutation | NM_000038.5 | 28.32% |
| SHPRH | p.G213A (c.638G>C) | Missense Mutation | NM_001042683.2 | 27.29% |
| PTP4A1 | p.H103Y (c.307C>T) | Missense Mutation | NM_003463.3 | 27.06% |
| H3C4 | p.R27P | Missense Mutation | NM_003530.3 | 26.99% |
| PIK3CG | p.G334S (c.1000G>A) | Missense Mutation | NM_002649.2 | 24.98% |
| LRP1B | p.H2813R (c.8438A>G) | Missense Mutation | NM_018557.2 | 24.26% |
| RICTOR | p.S1654C (c.4961C>G) | Missense Mutation | NM_152756.3 | 23.93% |
| ERCC6 | p.H210N (c.628C>A) | Missense Mutation | NM_000124.2 | 16.8% |
| ZFHX4 | p.M1724I (c.5172G>A) | Missense Mutation | NM_024721.4 | 15.64% |
| YWHAZ | p.E66Q (c.196G>C) | Missense Mutation | NM_001135699.1 | 6.76% |
| TAF15 | p.E323Q (c.967G>C) | Missense Mutation | NM_139215.2 | 1.67% |
| RYR2 | p.T3501N (c.10502C>A) | Missense Mutation | NM_001035.2 | 0.92% |
| GNA11 | p.K322N (c.966G>C) | Missense Mutation | NM_002067.2 | 0.7% |
| FAT3 | p.P1463L (c.4388C>T) | Missense Mutation | NM_001008781.2 | 0.69% |
