# Supplementary material for: Long-term survival after targeted therapy plus immunotherapy without chemotherapy in advanced gallbladder carcinoma: a case report and literature review
Source: Front Immunol. 2025 Sep 26;16:1629985. doi: 10.3389/fimmu.2025.1629985 (PMC12510835; doi:10.3389/fimmu.2025.1629985)
Supplement: Supplementary file 5 [file Table2.docx]

**Table S2.** Summary of advanced gallbladder carcinoma cases achieving favorable prognosis with targeted therapy and/or immunotherapy.

| **First author, year** | **Sex/age (years)** | **Tumor staging** | **Chemotherapy** | **TT/IO** | **ITB status** | **Surgery** | **Follow-up** |
| --- | --- | --- | --- | --- | --- | --- | --- |
| Kong *et al*, 2019^25^ | F/69 | T3N0M0  (Stage IIIA) | Gem+Cap, GEMOX | Nivolumab | PD-L1(+), MSS,  TMB-L | No | PFS more than 6 months |
| Prieto *et al*, 2019^26^ | M/44 | T3N0M1  (Stage IVB) | Cap+ Oxaliplatin | Trastuzumab | No | Yes | PFS more than 5 years |
| Li *et al*,  2020^27^ | F/61 | Stage IVB | Oxaliplatin+Cap | Pembrolizumab | PD-L1(-), MSS,  TMB-L | No | DFS more than 14 months |
| Rao *et al*, 2020^28^ | F/70 | Stage IVB | None | Camrelizumab Apatinib | PD-L1(-), MSS,  TMB-L | Yes | DFS more than 5 months |
| Sun *et al*, 2021^29^ | F/55 | T2N2M1  (Stage IVB) | GEMOX, AG | Crizotinib | No | Yes | Alive 9 months after diagnosis |
| Satyananda *et al*, 2021^30^ | M/59 | cT3N1M0  (Stage IIIB) | GC | Ipilimumab Nivolumab | NA | Yes | DFS at 10 months after surgery |
| Wu  *et al*, 2022^31^ | F/45 | T3N2M0  (Stage IIIA) | AG | Camrelizumab | NA | Yes | PFS at 14 months |
| Yi *et al, 2022^32^* | F/74 | Stage IVB | S-1 | Pembrolizumab | PD-L1(-), MSS,  TMB-L | Yes | Alive 32 months after diagnosis |
| Guo *et al, 2022^33^* | F/47 | T3N2M1  (Stage IVB) | Gem | Toripalimab Bevacizumab | MSS,  TMB-L | No | PFS more than 15 months |
| Wang *et al, 2022^34^* | M/67 | T3N0M0  (Stage IIIA) | Oxaliplatin | Camrelizumab Trastuzumab | TMB-H, | No | Alive more than 38 months after diagnosis |
| Zhang *et al*, 2023^35^ | F/60 | Stage IVB | GC | Lenvanitib Durvalumab | PD-L1(+), MSS | Yes | Alive 1 year after diagnosis |
| Wang *et al*, 2023^36^ | F/62 | T3N0M0  (Stage IIIB) | Cap | Tislelizumab Lenvatinib | TMB-H, MSS | No | None |
|  | M/79 | TxNxM1  (Stage IVB) | Cap | Tislelizumab Lenvatinib | TMB-L, MSS | No | None |
|  | F/63 | T3N0M0  (Stage IIIA) | GEMOX | Tislelizumab Lenvatinib | TMB-L, MSI | No | None |
|  | M/62 | T4NxM0  (Stage IVA) | S-1 | Tislelizumab Lenvatinib | TMB-L, MSS | No | Alive 14 months after diagnosis |
| Zhang *et al*, 2023^37^ | M/64 | T4N1M1  (Stage IVB) | S-1 | Tislelizumab | PD-L1(+), MSS,  TMB-L | Yes | Alive 2 years after diagnosis |
|  | F/69 | T4NxM1  (Stage IVB) | S-1 | Tislelizumab | PD-L1(-), MSS,  TMB-H | No | Alive more than 41 months after diagnosis |
|  | M/55 | T4N1M0  (Stage IVA) | S-1 | Tislelizumab | PD-L1(-), MSS,  TMB-H | Yes | PFS more than 18 months |
|  | F/53 | T3N2M1  (Stage IVB) | S-1 | Tislelizumab | PD-L1(-), MSS,  TMB-H | Yes | None |
|  | M/64 | T4N0M0  (Stage IVA) | S-1 | Tislelizumab | PD-L1(+) | Yes | None |
| Wang *et al*, 2023^38^ | F/58 | Stage IVB | Gem+Cap | Camrelizumab | PD-L1(+), MSS,  TMB-L | Yes | DFS at 14-month |
| Leong *et al*, 2024^39^ | M/39 | Stage IVB | Gem | Durvalumab | NA | Yes | DFS at 6-month |
| Wang *et al*, 2024^40^ | F/57 | T3N1M1  (Stage IVB) | mFOLFIRINOX | Toripalimab | TMB-H | No | Alive 2 years after diagnosis |
| Cui *et al*, 2024^41^ | F/46 | T3N0M0  (Stage IIIA) | GC | Lenvatinib: Toripalimab | NA | Yes | DFS at 13-months after surgery |
| Orlandi *et al*, 2024^42^ | M/62 | T4N2M0  (Stage IVB) | GC | Durvalumab | PD-L1(-), MSS, | Yes | None |
| Araki *et al*, 2025^43^ | F/68 | cT3N2M0  (Stage IVB) | GC | Durvalumab | PD-L1(-) | Yes | DFS at 8-months after surgery |
| Chen *et al*, 2025^44^ | M/68 | T3N1M0  (Stage IIIB) | GC | Durvalumab | NA | Yes | None |

*F* Female, *M* Male, *TT* Targeted therapy, *CR* Complete response, *IO* Immuno-Oncology, *ITB* Immunotherapy biomarkers, *GC* Gemcitabine cisplatin, *GEMOX* gemcitabine and oxaliplatin, *Gem* Gemcitabine, *Cap* Capecitabine, *DFS* Disease-free survival, *PFS* Progression free survival.
